# Ultrafast Charge and Exciton Diffusion in Monolayer Films of 9‐Armchair Graphene Nanoribbons

**DOI:** 10.1002/adma.202407796

**Published:** 2024-10-28

**Authors:** Sebin Varghese, Jake Dudley Mehew, Hai I. Wang, Michael Wuttke, Yazhou Zhou, Klaus Müllen, Akimitsu Narita, Aron W. Cummings, Klaas‐Jan Tielrooij

**Affiliations:** ^1^ Catalan Institute of Nanoscience and Nanotechnology (ICN2) CSIC and BIST, Campus UAB, Bellaterra Barcelona 08193 Spain; ^2^ Eindhoven University of Technology Den Dolech 2 Eindhoven 5612 AZ The Netherlands; ^3^ Max Planck Max Planck Institute for Polymer Research Ackermannweg 10 55128 Mainz Germany; ^4^ Debye Institute for Nanomaterials Science Utrecht University Princetonplein 1 Utrecht 3584 CC The Netherlands; ^5^ Organic and Carbon Nanomaterials Unit Okinawa Institute of Science and Technology Graduate University 1919‐1 Tancha, Onna‐son, Kunigami‐gun Okinawa 904‐0495 Japan

**Keywords:** charge mobility, excitons, graphene nanoribbons, spatiotemporal microscopy

## Abstract

Determining the electronic transport properties of graphene nanoribbons is crucial for assessing their suitability for applications. So far, this has been highly challenging both through experimental and theoretical approaches. This is particularly the case for graphene nanoribbons that are prepared by chemical vapor deposition, which is a scalable and industry‐compatible bottom‐up growth method that results in closely packed arrays of ribbons with relatively short lengths of a few tens of nanometers. In this study, the experimental technique of spatiotemporal microscopy is applied to study monolayer films of 9‐armchair graphene nanoribbons prepared using this growth method, and combined with linear‐scaling quantum transport calculations of arrays of thousands of nanoribbons. Both approaches directly resolve electronic spreading in space and time through diffusion and give an initial diffusivity approaching 200 cm^2^ s^−1^ during the first picosecond after photoexcitation. This corresponds to a mobility up to 550 cm^2^ V^−1^ s^−1^. The quasi‐free carriers then form excitons, which spread with a diffusivity of tens of cm^2^ s^−1^. The results indicate that this relatively large charge carrier mobility is the result of electronic transport not being hindered by defects nor inter‐ribbon hopping. This confirms their suitability for applications that require efficient electronic transport.

## Graphical abstract

Schematic representation of 9‐armchair graphene nanoribbons subjected to a pump pulse, which generates charge carriers. The spatial and temporal dynamics of these carriers are investigated using a probe pulse with variable time delay Δt and a controllable spatial offset.

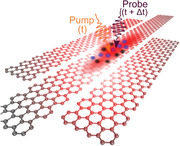

## 1 Introduction

Whereas 2D graphene is a gapless semimetal, in quasi‐1D graphene nanoribbons (GNRs) a bandgap is opened as a result of additional charge confinement. The properties of these graphene nanoribbons can be controlled by varying their width and edge structure. Graphene nanoribbons with zigzag edges tend to be metallic, while armchair edges give rise to increasingly large bandgaps for decreasing ribbon widths,^[^ ^1^, ^2^ ^]^ and increasingly 1D charge carriers. Besides these properties, they exhibit excitonic,^[^ ^3^, ^4^, ^5^, ^6^ ^]^ plasmonic,^[^ ^7^ ^]^ and topological^[^ ^8^ ^]^ properties. This makes graphene nanoribbons promising for electronic,^[^ ^9^ ^]^ optoelectronic,^[^ ^10^ ^]^ thermoelectric,^[^ ^11^ ^]^ spintronic,^[^ ^12^ ^]^ and quantum^[^ ^13^ ^]^ applications.

Early fabrication methods of GNRs took a top‐down approach, with lithographically defined ribbons with widths down to 10 nm,^[^ ^14^ ^]^ and solution‐processed graphite flakes that resulted in widths <10 nm.^[^ ^15^ ^]^ An alternative route to prepare GNRs is bottom‐up growth, where chemical synthesis using special molecular precursors produces GNRs with atomic precision both in width and edge termination, and with low defects.^[^ ^2^, ^16^, ^17^ ^]^ These organic chemistry approaches typically rely either on solution‐mediated or surface‐assisted reactions, and can produce GNRs with lengths exceeding 200 nm.^[^ ^4^ ^]^ Whereas the surface‐assisted approach initially required ultrahigh vacuum conditions, more recent demonstrations used lower vacuum conditions in chemical vapor deposition (CVD) setups.^[^ ^18^, ^19^ ^]^ While this approach leads to shorter lengths, it is compatible with industrial processing, which is crucial in order to move toward applications based on GNRs. In this work, we therefore focus on bottom‐up, CVD‐prepared GNRs, and aim to understand their charge transport properties, in order to evaluate their potential for practical applications.

An important parameter characterizing the suitability of GNRs for (opto)electronic applications is their charge mobility μ. As graphene‐derived materials, it is natural to expect high mobilities, especially in the case of smooth edges and low defects. A number of simulations and theoretical treatments have quantified the limits of GNR mobility,^[^ ^20^, ^21^, ^22^, ^23^ ^]^ as well as the performance of GNR‐based field effect transistors.^[^ ^24^, ^25^, ^26^, ^27^, ^28^ ^]^ With respect to the mobility of GNRs, edge disorder and structural defects were found to have the greatest impact, with electrostatic disorder arising from the substrate playing a secondary role.^[^ ^20^, ^22^ ^]^ In the defect‐free limit, electron–phonon scattering was predicted to limit the intrinsic mobility of narrow (≈1 nm) GNRs from a few hundred up to nearly 1000 cm^2^ V^−1^ s^−1^.^[^ ^20^, ^23^ ^]^ Most theoretical studies of transport in GNRs, however, have been limited to a single ribbon, while bottom‐up growth processes yield dense arrays of mostly aligned GNRs, separated into overlapping domains.^[^ ^29^ ^]^

In terms of experimental efforts to understand the electronic transport properties of GNRs, a common approach has been to fabricate a field‐effect transistor, where the GNR forms the channel, and measure the gate‐dependent current under a source‐drain bias voltage. Such measurements yielded estimated mobilities on the order of 100–200 cm^2^ V^−1^ s^−1^ for top‐down chemically derived GNRs.^[^ ^15^ ^]^ For GNRs that were grown inside pre‐etched trenches in hexagonal BN, field‐effect mobilities exceeding 1000 cm^2^ V^−1^ s^−1^ were obtained. However, for CVD‐grown GNRs, the obtained charge mobilities from electrical measurements are much lower: 10^−6^ to 10^−4^ cm^2^ V^−1^ s^−1^,^[^ ^18^ ^]^ and 10^−2^ cm^2^ V^−1^ s^−1^.^[^ ^30^ ^]^ The reason for these much lower values is the smaller ribbon length, which has made it impossible so far to fabricate a field‐effect transistor out of a single ribbon. As a result, measurements were performed on thin films of ribbons, rather than on a single GNR. The obtained mobility therefore does not reflect the intrinsic GNR mobility, and is instead thought to be governed by the hopping between different ribbons and transport across domains.

Contact‐free optical techniques offer benefits over electrical field‐effect measurements. In particular, they don't require complex nanofabrication of metallic contacts, and don't suffer from artifacts and uncertainties caused by contact resistance. Furthermore, they can provide the microscopic properties, rather than macroscopic properties that correspond to the full device size, possibly including grain boundaries and ribbon–ribbon intersections. Finally, optical techniques can probe transport properties away from band edges, which can be valuable for optoelectronic applications. A promising technique in this regard is optical‐pump terahertz‐probe spectroscopy. Measurements with this technique gave a mobility of ≈350 cm^2^ V^−1^ s^−1^ for 9‐atom wide armchair GNRs (9‐aGNRs).^[^ ^31^ ^]^ This technique is sensitive to microscopic transport properties, because of the limited average distance traveled by charges during interaction with the short pulses. However, this technique typically requires samples with large sizes, and averages over transport in ribbons with different orientations, limited by the mm‐sized diffraction limit of the THz probe. The obtained frequency‐resolved photoconductivities are furthermore often complex and deviate from the Drude response in the first few ps where free carriers populate in GNRs.^[^ ^6^ ^]^ Because of this, inferring the charge carrier mobility from descriptions with multi‐parameter alternative transport models is nontrivial. Finally, neither field‐effect transistor measurements nor THz photoconductivity measurements are sensitive to the transport properties of the charge‐neutral excitons that are known to form in GNRs.^[^ ^3^, ^4^, ^5^, ^6^ ^]^

Here we overcome these limitations and uncertainties by exploiting the all‐optical technique of spatiotemporal microscopy, which gives access to the intrinsic charge diffusivity without the need for any known material parameters. The diffusivity reflects how photo‐induced charges spread out in space as a function of time, and is directly related to charge mobility. In addition, this technique enables us to determine how the diffusivity changes as a function of time with sub‐picosecond time resolution. We apply this technique to a monolayer film of CVD‐grown 9‐aGNRs and compare our results with large‐scale quantum transport simulations of an array of 9‐aGNRs. The combined experimental‐theoretical results provide the intrinsic diffusivity of quasi‐free particles in CVD‐grown ribbon networks, which peaks at almost 200 cm^2^ s^−1^ during the first picosecond after photoexcitation, which corresponds to a mobility of 550 cm^2^ V^−1^ s^−1^. We also obtain the diffusivity of excitons, which is on the order of a few tens of cm^2^ s^−1^.

## 2 Results and Discussion

### 2.1 Experimental Results

We prepared monolayer films of 9‐aGNR using a CVD process with 3′,6′‐dibromo‐1,1′:2′,1″‐terphenyl (DBTP) as the monomeric building block, followed by a simplified wet‐etch transfer process with polyacrylamide (pAAM) as support. We transferred the monolayer films onto silicon nitride substrates. Details on the growth and transfer processes are described in Ref. [32]. This work also showed through field‐effect transistor measurements that the 9‐aGNR samples are undoped at zero gate voltage. The width of similar 9‐aGNRs was confirmed in Ref. [31] to be ≈1.2 nm, corresponding to 9 carbon atoms. Atomic Force Microscopy and Scanning Tunneling Microscopy images of similarly prepared samples show ribbon lengths up to 20 nm, closely packed in domains with a typical size of ≈30 nm.^[^ ^31^ ^]^ The ribbon length distribution of similar samples was also studied, see Ref. [29]. We studied two separate samples of monolayer 9‐aGNR films on silicon nitride, which gave very similar results. All results shown here are obtained from the same sample. The Raman spectrum of this sample is nearly identical to the Raman spectra obtained for 9‐aGNR samples in Ref. [32] (see Figure S1, Supporting Information).

**Figure 1:**
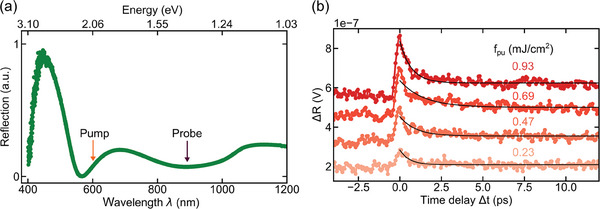
Absorption and decay dynamics of graphene nanoribbons. a) Reflection spectroscopy measurements of graphene nanoribbons, revealing enhanced absorption peaks at ≈600 and 900 nm. b) Temporal dynamics of graphene nanoribbons for various pump fluences *f*_pu_, with the solid (black) line representing a single exponential fit to the data for delay times greater than zero (Δ*t* > 0).

In order to obtain the true microscopic charge mobility of 9‐armchair GNRs, we use the technique of ultrafast spatiotemporal microscopy. This technique combines femtosecond temporal resolution with nanometer spatial accuracy by spatially scanning a probe pulse over a fixed pump pulse (see Experimental section and Figure S2, Supporting Information). The probe pulse has a variable offset with respect to the pump pulse in both time and space. As such, this technique provides very direct measurements of diffusive behavior of energy, (quasi‐)particles and heat, as recently reviewed.^[^ ^33^ ^]^ We use pump pulses with a wavelength of 600 nm (photon energy of 2 eV), pulse duration of ≈150 fs, and spot size <1 µm, which generate photoexcited carriers every 13 ns (the inverse of our laser repetition rate). The probe pulses have a wavelength of 900 nm (photon energy of 1.4 eV) and a similar pulse duration and spot size as the pump pulses. We monitor the pump‐induced change in reflection Δ*R* of the probe pulses at variable pump‐probe time delay Δ*t* and spatial offset Δ*x*, and quantify this through the voltage response of a photodetector. We chose the pump and probe wavelengths based on linear reflection spectroscopy measurements, which show enhanced absorption around these wavelengths (see **Figure** **1**a). For more information on the experiment, see the Experimental Section.

We first examine the ultrafast dynamics of the sample by overlapping the pump and probe spots on the sample, which means that Δ*x* = 0, and varying the time delay Δ*t* (see Figure 1b). We observe that the signal increases with pump fluence. The observed ultrafast rise and picosecond decay of the transient reflectivity signal are in agreement with earlier time‐resolved measurements on the same type of nanoribbons,^[^ ^4^, ^31^ ^]^ and reflect the transient generation of photoexcited charge carriers and their subsequent relaxation into excitons. We describe the dynamics with a single exponential decay function and obtain a decay time of ≈1 ps (see Table S1, Supporting Information). This decay time reflects the decay of quasi‐free charges into excitons.^[^ ^34^ ^]^ We verified that the signal at Δ*x* = 0 depends linearly on the pump fluence, which means that the spatial profiles will represent carrier densities (see Figure S3, Supporting Information).

We also observe a signal before time‐zero, which reflects a long‐lived heating signal: A small negative time delay also corresponds to a pump‐probe delay of ≈13 ns, which is the time between sequential pump (and probe) pulses as defined by the laser repetition rate. The photoexcited charge carriers transfer their energy to the lattice as phonon heat on a picosecond timescale, and the lattice does not have enough time to cool before the next pump pulse arrives, 13 ns after the previous one. Such a pre‐time‐zero signal can, in certain cases, be used to study lattice heat transport.^[^ ^35^ ^]^ In this work, we focus on the signal during the first few picoseconds, as this reflects the electronic properties of the GNRs.

**Figure 2:**
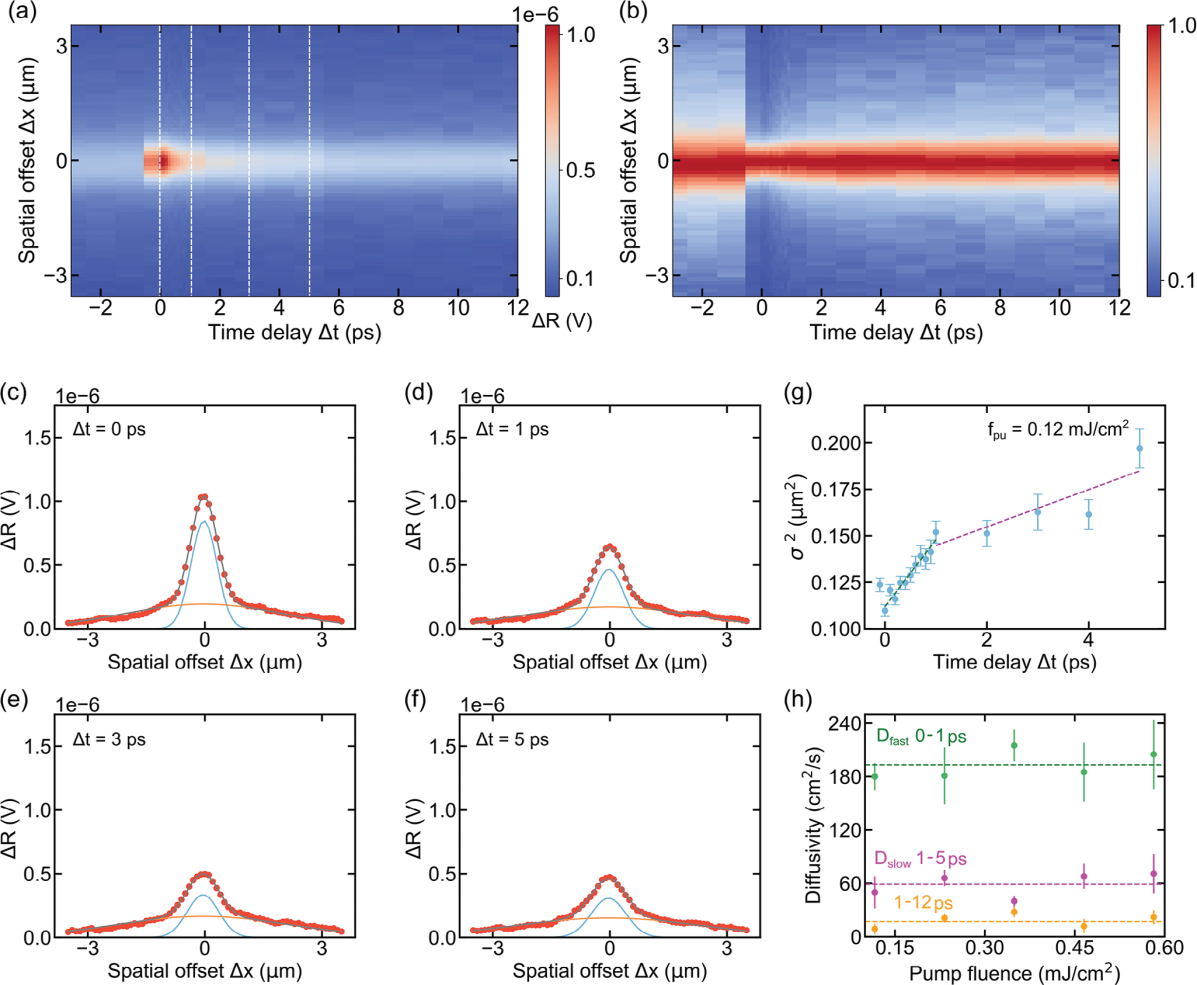
Spatiotemporal measurements and extracted diffusivity. a) Spatiotemporal dynamics of the transient reflection signal (units in volts V). The probe beam is scanned over the pump beam (1D scan across spot center, vertical axis) as a function of pump‐probe delay (horizontal axis) at a pump fluence of 0.12 mJ cm^−2^. The color scale indicates the transient reflection signal Δ*R*. b) Normalized spatiotemporal dynamics for data presented in (a). The signal broadening as a function of time delay indicates diffusion. c–f) Spatial profiles (scatters) with double Gaussian descriptions (solid lines) for selected time delays (dashed lines of panel (a)). g) Squared width evolution of the spatial profile, extracted from the narrow Gaussian component of the fits at each time delay. The error bar shows the 68% confidence interval of the fits. The slopes (dashed lines) give the initial fast diffusion and subsequent slow diffusion coefficients. We find fast diffusion of 200 cm^2^ s^−1^ within 1 picosecond, followed by a slow diffusion of 60 cm^2^ s^−1^ (1–5 ps), and ≈20 cm^2^ s^−1^ (1–12 ps). h) Extracted diffusion coefficients for pump fluences in the 0.12–0.58 mJ cm^−2^ range. The dashed lines represent average values of the experimentally obtained diffusivities.

In order to study electronic diffusion, we vary both Δ*t* and Δ*x*, and obtain the spatiotemporal spreading of the photoexcited signal. The measured spatiotemporal dynamics are shown in **Figure** **2**a. From the normalized signal in Figure 2b, it is clear that the narrow photoexcited distribution at time‐zero starts spreading spatially as a result of diffusion. This is also evident from the spatial profiles at a Δ*t* of 0 ps (see Figure 2c), 1 ps (see Figure 2d), 3 ps (see Figure 2e), and 5 ps (see Figure 2f). The profile at 5 ps is the broadest one (see also Figure S4, Supporting Information). At all delays, there is a background signal that is spatially very broad, which we ascribe to long‐lived lattice heat spreading. We therefore describe these profiles using two Gaussian profiles – one broad profile that reflects the lattice heat; and one narrower profile that reflects photoexcited carriers. We note that due to the broad temperature profile, the temperature is relatively constant in the region where photoexcited carriers diffuse. Furthermore, phonon scattering typically does not play a role in the charge transport of graphene at room temperature,^[^ ^36^, ^37^ ^]^ which means that a variation in phonon temperature will not affect charge mobility and diffusivity.

The obtained widths that correspond to lattice heat do not vary with delay time, and have a full width at half maximum (FWHM) of ≈4 µm (see Figure S5, Supporting Information). Since this profile corresponds to heat that accumulates over multiple pump pulses, it makes sense that it does not depend on the pump‐probe delay time. The widths of the electronic excitation do change with delay time, see Figure 2g. Specifically, we observe a fast linear increase of the squared width as a function of time delay during the first ≈1 ps, followed by a slower increase. Remarkably, we observe charge diffusion over distances larger than 100 nm, which is larger than the typical GNR length of 20 nm. This suggests that charge transport between neighboring GNRs with some overlap in these films is efficient, and therefore that inter‐ribbon hopping does not significantly limit the diffusivity and mobility.

We find associated diffusivities of ≈200 cm^2^ s^−1^ for the first picosecond, and ≈60 cm^2^ s^−1^ for the next few picoseconds (1–5 ps), see Figure 2h. Since we find a decay time on the order of a picosecond, the initial fast diffusion component reflects quasi‐free photoexcited charge carriers. The slower diffusion component corresponds to the diffusion of excitons. We performed spatiotemporal measurements also with varying pump laser fluence and obtained similar trends and diffusivity values (see Figure 2h; Figure S6, Supporting Information). The sharp transition to a much less steep slope, occurring at a delay of ≈1 ps, suggests a crossover between different diffusing species. In the case of these GNRs, this corresponds to quasi‐free charges and excitons. Some datasets suggest that a short‐lived apparent negative diffusion is present during the transition from fast diffusion of quasi‐free charges to slower diffusion of bound excitons. Similar behavior has been observed during the transition from fast‐diffusing electronic heat to slow‐diffusing phononic heat in gold.^[^ ^38^, ^39^ ^]^

Regarding the exciton diffusion occurring beyond a delay of ≈1 ps, we note that if we consider a range of 1–12 ps, the diffusivity is significantly lower than when considering the range up to 5 ps: ≈20 cm^2^ s^−1^ instead of ≈60 cm^2^ s^−1^ (see Figure 2h). This suggests a decreasing exciton diffusivity with increasing time delay. An explanation for this could be diffusion‐limited recombination, where fast diffusing pairs recombine more quickly than slowly diffusing pairs. Such a process of diffusion‐limited recombination was unraveled by pump‐probe measurements on single‐walled carbon nanotubes,^[^ ^40^ ^]^ which is a material system that closely resembles the GNRs that we study here.

### 2.2 Theoretical Results

In order to understand these experimental observations, we simulate the bulk transport properties of GNR arrays. This requires efficient simulation methods that can handle the atomistic characteristics of each ribbon, the detailed structure of its overlap with neighboring domains, while also reaching experimental transport length scales. We use a linear‐scaling quantum transport method to simulate quantum transport in GNR arrays. Specifically, we use an efficient real‐space approach that allows for the study of large‐area disordered systems containing many millions of atoms.^[^ ^41^ ^]^ The central quantity calculated in this approach is the mean‐square displacement (MSD) of an initial electronic state at a given energy *E*,

(1) $$X^{2}\left(E,t\right)=\frac{\left\langle \psi_{X}\left(t\right)\left|\delta \left(E-\hat{H}\right)\right|\psi_{X}\left(t\right)\right\rangle }{\left\langle \psi \left(0\right)|\delta \left(E-\hat{H}\right)|\psi \left(0\right)\right\rangle }$$

where $|\psi_{X}\left(t\right)\rangle =[\hat{X},\hat{U}\left(t\right)]\left|\psi (0)\right\rangle$, $\hat{X}$ is the position operator, $\hat{U}\left(t\right)=\exp\left(-i\hat{H}t/\hbar \right)$ is the time evolution operator, $\rho \left(E\right)=\frac{2}{A}\left\langle \psi \left(0\right)|\delta \left(E-\hat{H}\right)|\psi \left(0\right)\right\rangle$ is the density of states (DOS), *A* is the sample area, and $\hat{H}$ is the Hamiltonian of the system. In this approach the initial state |ψ(0)⟩ is taken to be a random phase state, which allows for efficient evaluation of ensemble‐averaged transport properties at an arbitrary energy *E*.^[^ ^41^, ^42^ ^]^

**Figure 3:**
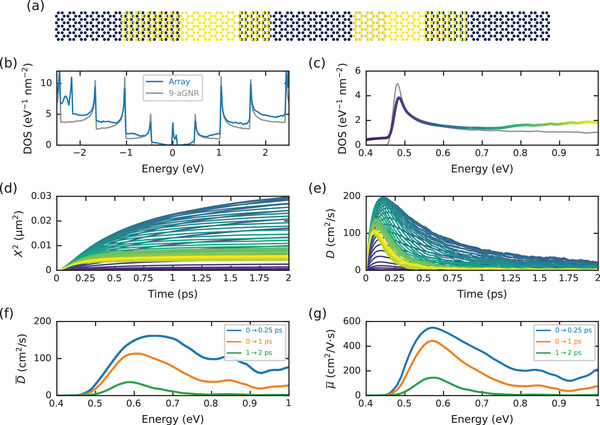
Simulation results of charge transport in arrays of 9‐aGNRs. a) Representation of the simulated system, consisting of an array of 9‐aGNRs, each with a random length and a random overlap with its neighbors. b) Density of states of the 9‐aGNR array (blue line) and of a single ideal 9‐aGNR (gray line). c) Zoom of the density of states around the lowest conduction subband. Here the DOS of the array has been colored according to its energy, to correspond to the colors of the curves in the next two panels. d) Time‐dependent mean square displacement, where the color of each curve corresponds to the energy scale in panel (c). e) Time‐dependent diffusivity, with the same color scale. f) Time‐averaged diffusivity, where the averages have been taken over the time ranges indicated in the legend. g) Time‐averaged mobility of the GNR array.

A representation of the system we simulate is shown in **Figure** **3**a. It consists of a linear chain of 9‐aGNRs, each with a random length and a random amount of overlap with its neighbors. The length of each ribbon is determined according to the experimental distribution (bromine precursor) shown in Figure 3c of Ref. [29], with an average length of 22 nm, and the overlaps are randomly chosen in the range 4 ± 2 nm. To achieve good statistical averaging of the transport properties, we consider a total of 200 000 GNRs in our simulations. To accurately describe both the isolated ribbons and the overlap regions, we use a tight‐binding Hamiltonian, derived from first principles calculations, that accurately describes the electronic properties of both single‐layer and bilayer graphene.^[^ ^43^ ^]^

The density of states of the GNR array is shown in Figure 3b. The gray line shows the DOS of a single perfect 9‐aGNR, with characteristic van Hove singularities at the band edges and a band gap of ≈1 eV.^[^ ^44^ ^]^ The blue line is the DOS of the GNR array, which exhibits extra states below the band edges, as well as the formation of localized states in the middle of the gap. These states are attributed to the zig‐zag ends of the finite ribbons, as well as the overlap regions, while the splitting of the peak at 0 eV arises from the interlayer coupling between overlapping ribbons (see Figures S7 and S8, Supporting Information). Meanwhile, the DOS is unchanged above the band edge, suggesting these states remain associated with carriers in the non‐overlapping portion of the GNRs. In Figure 3c we plot a zoom of the DOS around the lowest conduction subband. Given the photon energy of the optical probe (≈1.4 eV) pulses, this is the approximate energy range over which we expect transport to occur in the experiments. The DOS of the array has been progressively colored according to its energy, to correspond to the color of the time‐dependent curves in panels (d) and (e).

In Figure 3d we plot the time‐dependent MSD over the range of energies depicted in panel (c). At each energy, the MSD exhibits an initial fast increase associated with the ballistic regime of transport, a transition to a diffusive regime once scattering at the ribbon interfaces sets in, and eventually saturation as localization effects begin to dominate. In agreement with the experimental results, the simulations also indicate charge spreading well beyond the length of individual ribbons. This supports our notion that inter‐ribbon hopping does not strongly limit charge transport.

For low energies below the band edge (purple curves) and high energies below the second subband (yellow curves), transport is strongly localized, with the MSD quickly saturating to a fixed value after a few hundred fs. As highlighted in the DOS, these energies are characterized by states arising from the ends of the GNRs and their overlaps with their neighbors. Meanwhile, above the band edge (blue curves) electrons continue to spread during the entire simulation time scale, indicating robust transport at these energies.

These features are more easily seen in Figure 3e, where we plot the time‐dependent diffusivity at each energy, $D\left(E,t\right)=\frac{1}{2}\partial X^{2}/\partial t$. Here one can clearly see the initial fast increase associated with ballistic transport, the transition to diffusion as *D* saturates, and the onset of localization with the subsequent decrease of *D* with time. For low and high energies, transport is strongly localized and *D* → 0 after a few hundred fs, while above the band edge *D* remains finite over the entire simulation time scale. The simulations thus indicate that transport is dominated by carriers above the edge of the lowest subband, which is associated with transport through the pristine GNR regions. Meanwhile, transport is strongly inhibited near the subband edges, where states associated with the overlapping regions of the GNRs appear to dominate. The simulations thus suggest that transport measurements in a field‐effect geometry with the chemical potential tuned to the band edge would show lower mobilities.

In Figure 3e we have calculated the instantaneous diffusivity at each time step. Meanwhile, in the experiments the fast and slow diffusivity in Figure 2h were extracted by fitting the slope of the MSD over a finite time range. Thus, to provide a more quantitative connection between the experiments and the simulations, in Figure 3f we plot the time‐averaged diffusivity $\bar{D}$, where the average is taken over short, intermediate, and long time scales. The short time scale, from 0 to 250 fs, encompasses the initial fast increase of MSD associated with the ballistic regime and the transition to diffusive transport. Over this time scale, $\bar{D}$ peaks at ≈160 cm^2^ s^−1^ above the band edge, similar to that seen experimentally. Meanwhile, in the long time scale from 1 to 2 ps, $\bar{D}$ is suppressed at all energies outside the middle of the band, where it peaks at ≈35 cm^2^ s^−1^, also in line with the measurements.

Finally, in panel (g) we compute the corresponding time‐averaged mobility using the generalized Einstein relation, $\bar{\mu }=e\bar{D}\cdot \left(\partial n/\partial E\right)/n$, where *n* is the carrier density, obtained via direct numerical integration of the DOS. Here we see that in the short time scale before the slower diffusion regime kicks in, the mobility reaches $\bar{\mu }\approx 550$ cm^2^ V^−1^ s^−1^, while in the long time scale it peaks at $\bar{\mu }\approx 145$ cm^2^ V^−1^ s^−1^.

In our simulations there are no other defects and transport is determined entirely by hopping between adjacent ribbons. Thus, the angle of rotation between overlapping ribbons may have a significant impact on the overall transport properties of the GNR array. To examine this, we have also run simulations where the angle between each pair of overlapping ribbons is randomly distributed over some range (see Figure S9, Supporting Information). For small‐angle rotations between adjacent ribbons (±2°) we find a minimal impact on charge transport. Meanwhile, perpendicular rotations between adjacent ribbons (90° ± 2°), which represent the worst‐case scenario, exhibit a strong suppression of transport. These results indicate that inter‐ribbon transport remains efficient within domains of well‐aligned GNRs. Meanwhile, transport would be strongly limited between misaligned or rotated GNR domains.

## 3 Conclusion

In summary, we have used a combination of ultrafast spatiotemporal microscopy and numerical simulations to investigate the intrinsic nature of charge transport in bottom‐up‐grown arrays of 9‐armchair GNRs. From the measurements, we see an initial fast diffusion of quasi‐free carriers with a diffusivity *D* ≈ 200 cm^2^ s^−1^, which is similar to the diffusivity we find from the simulations during the first few hundred femtoseconds. This corresponds to a mobility of ≈550 cm^2^ V^−1^ s^−1^, and is close to the 350 cm^2^ V^−1^ s^−1^ that was found using THz measurements.^[^ ^31^ ^]^ We note that a recent spatiotemporal study showed that the diffusivity of charge carriers in high‐quality exfoliated graphene that was encapsulated by hexagonal BN is ≈2000 cm^2^ s^−1^, corresponding to a mobility ≈40 000 cm^2^ V^−1^ s^−1^.^[^ ^45^ ^]^ The mobility of CVD‐grown graphene that is not encapsulated by hexagonal BN will typically be around ten times lower, which means that the diffusivity of non‐encapsulated graphene is on the order of a few hundred cm^2^ s^−1^, i.e., the same range as for the 9‐aGNR samples.

Here we would like to comment on the contrast between this work and prior ones that observed extremely low mobilities (<0.1 cm^2^ V^−1^ s^−1^) in bottom‐up GNR arrays.^[^ ^18^, ^30^ ^]^ In those works, the mobility was extracted from the measurement of field‐effect transistors with channel lengths ranging from 500 nm up to 10 µm. On those length scales, the measurements are likely probing transport across multiple GNR domains. Within each domain, the ribbons may be aligned and exhibit good transport, but cracks or voids between domains, as well as large misalignment angles, would lead to very high inter‐domain resistance and low overall mobility. Meanwhile, our measurements and simulations probe spreading of the carrier population up to at most a few hundred nm. On these length scales, we posit that we are measuring primarily intra‐domain transport, with our results indicating good hopping between aligned GNRs and high intra‐domain mobility.

Interestingly, the simulations correspond to transport along the graphene nanoribbons, while the experiments are performed on non‐aligned ribbons. The observation that the experimental and theoretical diffusivities are in agreement suggests that the experiment mainly probes transport along the ribbon.^[^ ^46^ ^]^ The reason for this is that ribbons that are not aligned with the pump and probe polarization are not excited and probed efficiently. The diffusion for time delays beyond 1 ps corresponds to excitons, rather than quasi‐free charges. The experimentally obtained exciton diffusivity of a few tens of cm^2^ s^−1^ is similar to the diffusivity of excitons in 2D monolayers of layered semiconductors, such as WS_2_,^[^ ^47^ ^]^ MoSe_2_,^[^ ^48^ ^]^ MoS_2_,^[^ ^49^ ^]^ and WSe_2_.^[^ ^50^ ^]^ These are monocrystalline materials prepared by exfoliation, which typically have low defect densities, on the order of 10^9^ cm^−2^.^[^ ^51^ ^]^

The observed charge mobility and exciton diffusivity suggest that the defect density of these GNRs is low. This is corroborated by the simulation results, where the only source of disorder is from the finite length of the GNRs and the overlaps with their neighbors. Thus, the diffusion of carriers in these samples appears to be limited only by the overlapping regions of the individual GNRs, or by domain boundaries, and not by defects and disorder within the GNRs themselves. In this regime, we suggest that mobilities may be improved by increasing the average GNR length and domain size, or via careful control of the overlapping regions between neighboring GNRs through post‐processing. The insights obtained from the combined experimental theoretical approach are crucial for optimizing materials for high‐performance applications in nanoelectronics, optoelectronics, and other advanced technological fields.

## 4 Experimental Section

### Spatiotemporal Measurements

To perform spatiotemporal measurements, a FLINT laser operating at 76 MHz, producing pulses centered at 1030 nm with an approximate temporal resolution of 150 fs was employed. Most of the laser power was directed to an optical parametric oscillator (OPO), which generates a variable signal output from 1320 to 2000 nm. The pump beam, at 600 nm, was the third harmonic of the 1800 nm signal output and was directed through a Newport DL255 delay line and a mechanical chopper (modulation frequency f_mod_ = 4.73 kHz). The probe beam, at 900 nm, was the second harmonic of the signal output. A scanning mirror system (Optics in Motion OIM101) was used to control the probe's direction. Both beams were combined using a dichroic mirror and focused onto sub‐micrometer spots with a microscope objective lens (numerical aperture NA = 0.65). The reflected probe beam was detected with a silicon photodiode and demodulated at 4.73 kHz using a lock‐in amplifier (Zurich MFLI). Schematics of the setup can be found in Figure S2 (Supporting Information) and Ref. [35].

## Conflict of Interest

The authors declare no conflict of interest.

## Supporting information

Supporting Information

## Data Availability

The data that support the findings of this study are available from the corresponding author upon reasonable request.
