# Supplementary material for: Ultrafast Charge and Exciton Diffusion in Monolayer Films of 9‐Armchair Graphene Nanoribbons
Source: Adv Mater. 2024 Oct 28;36(50):2407796. doi: 10.1002/adma.202407796 (PMC11636163; doi:10.1002/adma.202407796)
Supplement: Supplementary file 1 — Supporting Information [file ADMA-36-2407796-s001.pdf]

ADVANCED MATERIALS

Supporting Information

for *Adv. Mater.*, DOI 10.1002/adma.202407796

Ultrafast Charge and Exciton Diffusion in Monolayer Films of 9-Armchair Graphene Nanoribbons

Sebin Varghese, Jake Dudley Mehew, Hai I. Wang, Michael Wuttke, Yazhou Zhou, Klaus Müllen, Akimitsu Narita, Aron W. Cummings and Klaas-Jan Tielrooij**

Ultrafast Charge and Exciton Diffusion in Monolayer Films of 9-Armchair Graphene Nanoribbons

Sebin Varghese[1,2] *Jake Dudley Mehew*[1] *Hai I. Wang*[3,4] *Michael Wuttke*[3] *Yazhou Zhou*[3] *Klaus Müllen*[3] *Akimitsu Narita*[3,5] *Aron W. Cummings*[1,@] *Klaas-Jan Tielrooij*[1,2,@]

S. Varghese, J.D. Mehew, A.W. Cummings, K.J. Tielrooij
[1] Catalan Institute of Nanoscience and Nanotechnology (ICN2), CSIC and BIST, Campus UAB, Bellaterra, 08193 Barcelona, Spain
S. Varghese, K.J. Tielrooij
[2] Eindhoven University of Technology, Den Dolech 2, 5612 AZ, Eindhoven, the Netherlands
Email Address: k.j.tielrooij@tue.nl
H.I. Wang, M. Wuttke, Yazhou Zhou, K. Müllen, A. Narita
[3] Max Planck Max Planck Institute for Polymer Research, Ackermannweg 10, 55128 Mainz, Germany
H.I. Wang
[4] Debye Institute for Nanomaterials Science, Utrecht University, Princetonplein 1, 3584 CC Utrecht, The Netherlands
A. Narita
[5] Organic and Carbon Nanomaterials Unit, Okinawa Institute of Science and Technology Graduate University, 1919-1 Tancha, Onna-son, Kunigami-gun, Okinawa 904-0495, Japan

[@] Correspondence to aron.cummings@icn2.cat, k.j.tielrooij@tue.nl

1 Raman characterization

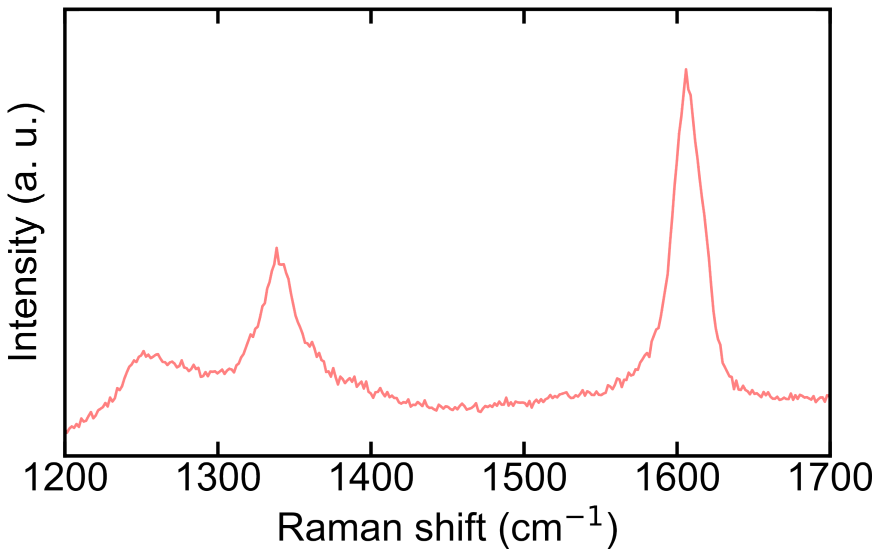

Figure S1: **Raman measurements.** Raman spectrum of our sample, which reveals three main peaks at 1260, 1340, and 1607 cm^{-1}. This is very similar to the results in Ref. [1], where the peaks of 9-aGNR films, which were prepared in the same way as our sample, were observed at 1251, 1338, and 1602 cm^{-1}.

2 Experimental setup

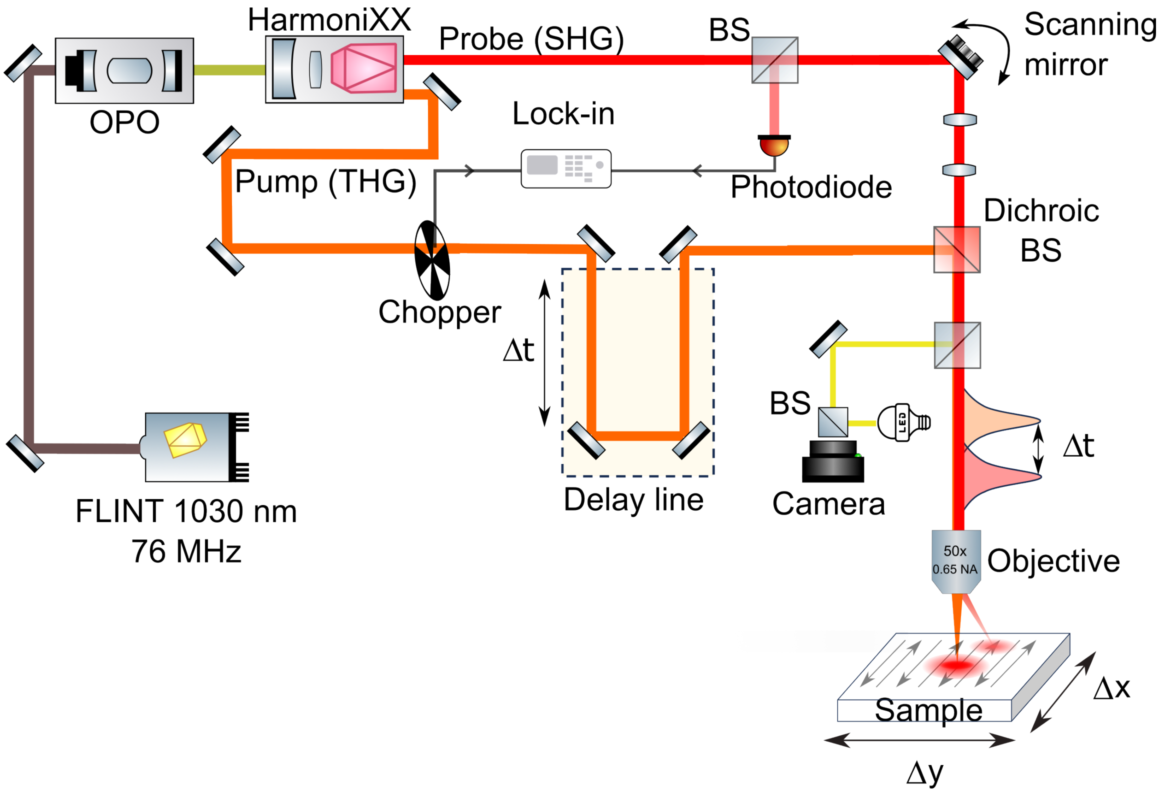

Figure S2: **Spatiotemporal experimental setup.** Schematics of the spatiotemporal experimental setup. A detailed description of the experiment (and setup) can be found in the main text. OPO: optical parametric oscillator, BS: beam splitter, LED: light emitting diode, THG: third harmonic generation, SHG: second harmonic generation, NA: numerical aperture.

Pump fluence (mJ/cm^2)	Decay time (ps)
0.12	0.6
0.23	1.0
0.35	1.4
0.46	0.7

Table S1: Decay time obtained from the exponential fit to the temporal dynamics shown in Fig. 1b of the main text.

3 Linearity with pump fluence

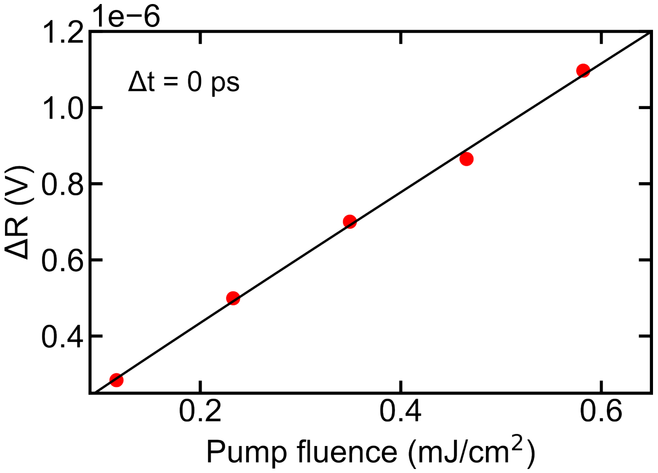

Figure S3: **Linearity with fluence.** Transient reflectivity signal ΔR at $\Delta t = 0$ ps (scatters) with pump fluence. The solid line is a power-law fit, yielding the exponent of fit to be 0.97, indicating that the relationship is very close to linear.

4 Normalized profiles

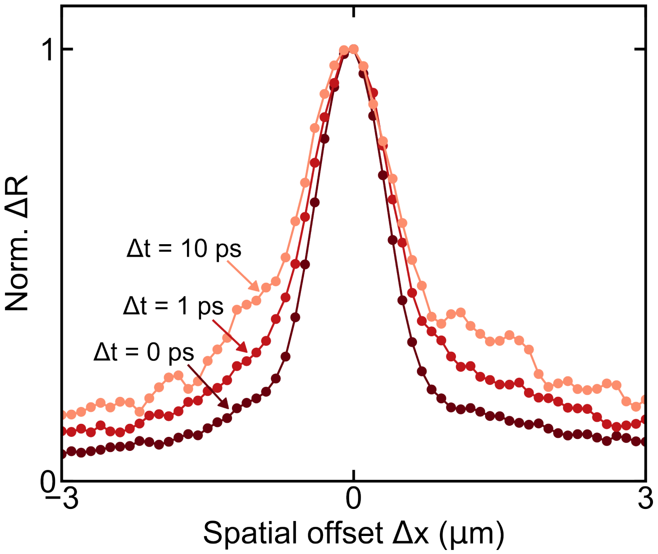

Figure S4: **Normalized profiles.** This normalization isolates the spatial broadening effect from the effect of decaying signal, and clearly shows broadening with time due to the diffusion.

5 Broad Gaussian width with time delay

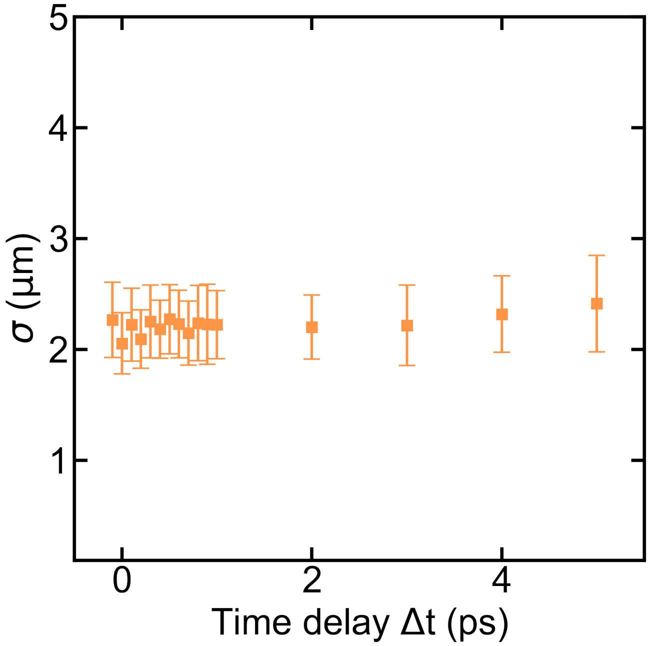

Figure S5: **Evolution of broad Gaussian width.** The width of the broad Gaussian remains nearly constant over the measured time delays. Pump fluence: 0.23 mJ/cm^2.

6 Evolution of squared width at varying pump fluence

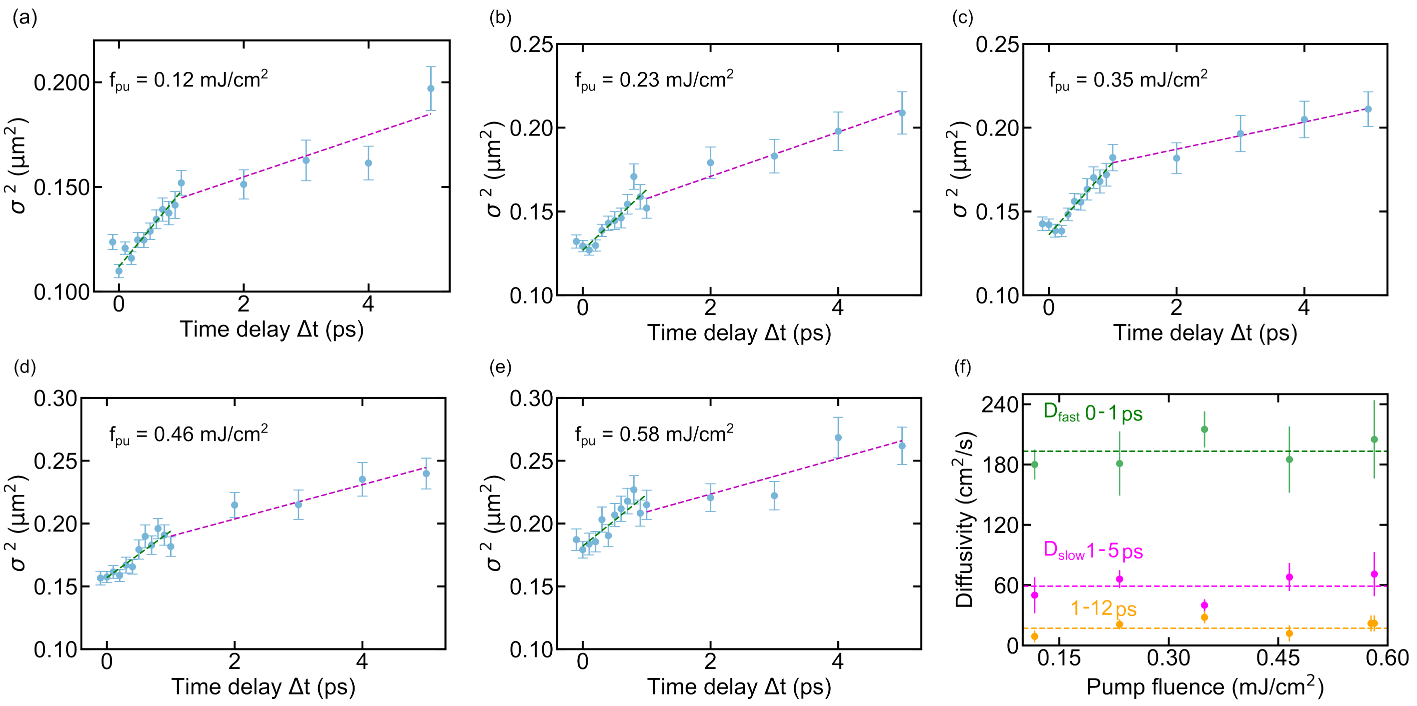

Figure S6: **Diffusion at varying pump fluence.** (a-e) Squared width evolution of the spatial profile, extracted from the narrow Gaussian component of the two Gaussian fits at each time delay for different pump fluences. The error bar shows the 68% confidence interval of the fits. Initial fast diffusion and subsequent slow diffusion coefficients are extracted by fitting slopes in the two regions. (f) Obtained fast and slow diffusivity with pump fluence. The dashed lines represent the average values of the measured diffusivities.

7 Splitting of mid-gap DOS peak

In Fig. 3(b) of the main text, the mid-gap (0 eV) peak in the density of states (DOS) is split. To explain the origin of this, we first explain the origin of the peak at 0 eV. Because the 9-aGNRs are finite in length, the ends of each ribbon consist of a zig-zag edge of 9 carbon atoms. This can be seen for example in Fig. 3(a) of the manuscript. In the DOS, the peak at 0 eV corresponds to states that are localized at these zig-zag edges. This can be seen in Fig. S7 below, where in panel (a) we show the DOS of an individual 9-aGNR with a length of 20 nm (the spikiness arises from the finite number of atoms in this finite-length ribbon), and in panel (b) the corresponding local density of states (LDOS) at 0 eV. The red color in the LDOS indicates a high concentration of electron population localized at the zig-zag ends of the ribbon.
Next, in the full GNR array we have a series of many ribbons that each overlap with their two adjacent neighbors, as indicated in Fig. 3(a) of the manuscript. In these overlapping regions, the interlayer interaction between ribbons splits the energy of the states localized on the zig-zag ends. To verify this, we have calculated the DOS of an array of 10,000 ribbons, with and without the interlayer interaction. As seen in Fig. S8 below, when there is no interaction between overlapping ribbons the low-energy peak in the DOS sits exactly at 0 eV (red curve). Meanwhile, turning on the interlayer interaction splits this peak (blue curve).

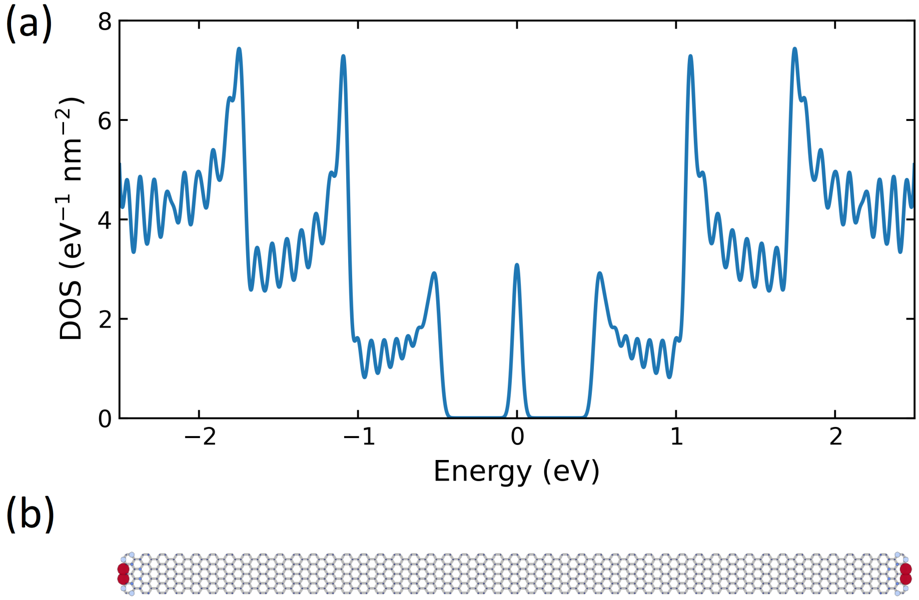

Figure S7: **DOS of individual 9-aGNR.** (a) DOS of an individual 9-aGNR with a length of 20 nm, with a peak at 0 eV. (b) LDOS at 0 eV, indicating a state localized at the zig-zag ends of the ribbon.

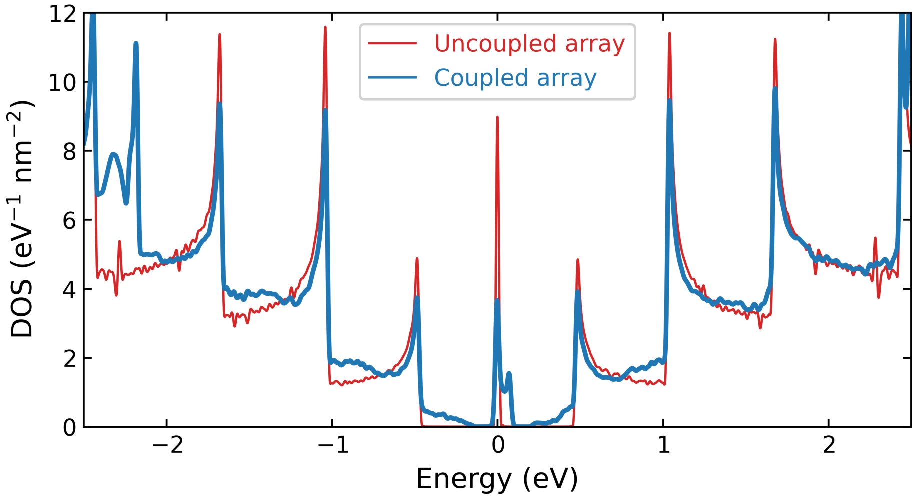

Figure S8: **DOS of a 9-aGNR array consisting of 10,000 ribbons.** The red curve shows the case when all ribbons are isolated, i.e., when there is no interlayer coupling between overlapping ribbons. The blue curve includes interlayer coupling.

8 Effect of ribbon rotation on transport

In our simulations in the main text, there are no defects and transport is determined entirely by hopping between adjacent ribbons. Thus, the angle of rotation between overlapping ribbons may have a significant impact on the overall transport properties of the GNR array. To examine this, we have run simulations where the angle between each pair of overlapping ribbons is randomly distributed over some range. First, we consider the low-angle case, where the angle between each pair of overlapping ribbons lies in the range $\theta = \pm 2°$. The resulting mobility is shown as the dashed lines in Fig. S9 below. Compared to the case where all ribbons are parallel ($\theta = 0°$, solid lines), we see that small-angle rotations have only a small impact on transport. Next, we consider the large-angle case with a rotation of $\theta = 90° \pm 2°$ between each pair of overlapping ribbons. This is shown as the dotted lines in Fig. S9. Here we see that perpendicular alignment between adjacent ribbons strongly suppresses charge transport.

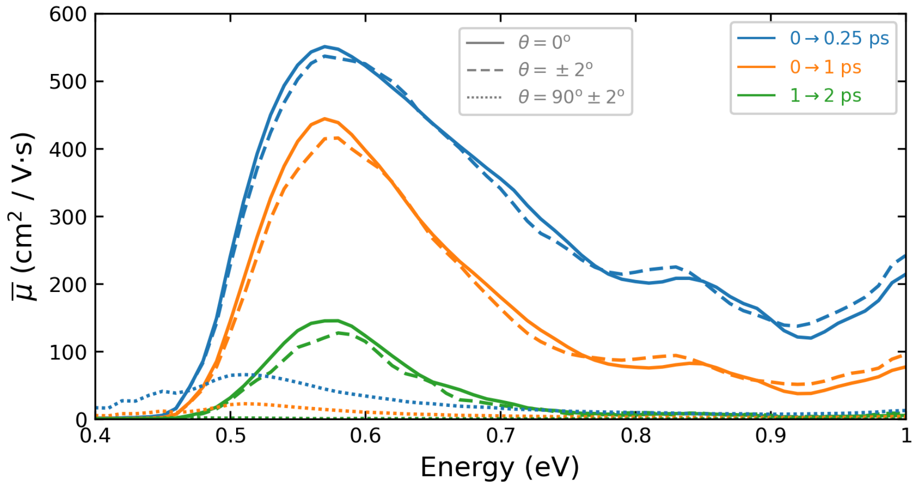

Figure S9: **Mobility of 9-aGNR with rotation angles.** Time-averaged mobility of the 9-aGNR array, for different rotation angles between neighboring ribbons. Solid lines are for all ribbons in parallel ($\theta = 0°$), dashed lines are for a rotation of $\theta = \pm 2°$, and dotted lines are for $\theta = 90° \pm 2°$.

References

[1] B. Jeong, M. Wuttke, Y. Zhou, K. Müllen, A. Narita, K. Asadi, *ACS Applied Electronic Materials* **2022**, *4*, 6 2667.
